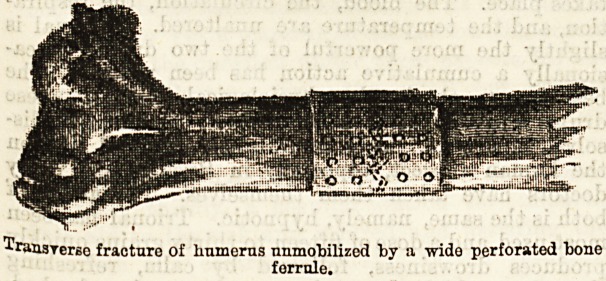# Progress in Surgery

**Published:** 1894-02-10

**Authors:** 


### PROGRESS IN SURGERY.

### ANTISEPTICS.

The antiseptic management of wounds has undergone many changes of late. Sir Joseph Lister[1] adduces some clinical experience to show the efficacy of carbolic acid in obtaining aseptic results in surgical wounds. He employs this in preference to corrosive sublimate; the staphylococcus pyogenes aureus—a very common cause of suppuration—is much more rapidly destroyed by carbolic acid than by the bichloride. His experiments have also shown that tubercle bacilli are more slowly destroyed by corrosive sublimate (1 in 1,000) than by carbolic acid 1 in 20. These bacilli need not be feared in sponges if they are kept for a considerable time in 1 in 20 carbolic solution. He treats the sponges by washing well with soap and water, and afterwards with soda; then thoroughly washes again with water, and finally, after drying, puts to steep in 1 in 20 carbolic solution till they are required for use. For private operations he places the sponges after use in a tank of water and allows them to putrefy. The fibrin which clings among the spores of the sponges becomes liquified by putrefaction. They can then be washed thoroughly clean of their fibrin, and the washing continued until the water is no longer red. They are then kept in 1 in 20 carbolic solution. This same 1 in 20 carbolic solution he uses for purifying instruments (which is more convenient than boiling in private practice), and for purifying the hands of the operator and skin of the patient. The length of time for which the instruments should be kept in solution depends upon the care with which they are washed before putting them away. Any which have teeth require to be brushed with a nail brush before they are dried, so that there may be no crust of dry blood upon them. If this has been done, the instruments require to be placed only a short time in the solution (during the administration of the anæsthetic, &c.) The skin of the patient should be purified for a few minutes by the action of the same solution; it is not needful to apply it for hours together, or to use soap and water. Carbolic acid has a powerful affinity for the epidermis, penetrating deeply into its substance; and it mingles with fatty materials in any proportion. Corrosive sublimate, on the other hand, cannot penetrate in the slightest degree into anything greasy, and therefore those who use corrosive sublimate require elaborate precautions in the way of cleansing the skin—with oil of turpentine, ether, soap and water, &c.—to remove the grease before the corrosive sublimate can act efficiently. Lister advises washing the sponges during the operation in 1 in 40 carbolic solution, and finally the wound with the same before it is closed, because it is impossible to be always quite certain that the assistants have been careful. For dressing the wound, in the absence of chemical antiseptics, dry substances, such as absorbent cotton wool or old linen (preferably boiled before use) are far better than anything kept permanently wet like water dressing. With dry dressing and other care referred to, complete primary union should be of very frequent occurrence, instead of a rarity as formerly. Although IODOFORM has little influence on the growth of bacteria outside the body, it is unquestionably true that it exercises a powerful antiseptic influence upon wounds by inducing chemical changes in the toxic products of the bacteria, not by direct action upon the bacteria. It remains for a long time unconsumed among the tissues, and is remarkably free from irritating properties. In operations upon the mouth or rectum, or when putrid sinuses are present, iodoform is of high value. In such cases, before applying it, the cut surface should be mopped with a solution of chlorate of zinc 40 to the ounce of water. In compound fractures iodoform cannot be dispensed with.

The use of carbolic in full strength upon extensive cut surfaces is advocated by Allis.[2] The method was first introduced and extensively employed by Gardner. After ligation of the bleeding vessels in operations such as amputation of the breast, carbolic acid crystals dissolved in sufficient water for solution are applied with a sponge to all parts of the cut surface. The tissues immediately turn white, the surface is then washed with sterilized water, and the edges approximated with provisions for drainage. This is necessary, as oozing takes place for twenty-four hours. Gardner claims for carbolic acid in officinal strength: 1. No systemic absorption attends its use (like dilute solutions), and hence no danger or shock. 2. It is a local anæsthetic, hence there is not as much pain after the operation. 3. It is in a measure a hæmostatic, acting especially upon the capillary vessels. In hydrocele he lays the sac open freely, and applies the acid to the tunica vaginalis, packs, and drains. Allis found it especially valuable in deep sinuses and pus tracts. Care must be taken that the acid does not come in contact with the skin. A new substance called Izal, a by-product in the process of coke formation, has recently been used by Bruce Clarke.[3] It exhibits very remarkable disinfectant properties, at the same time is non-irritant, and has no poisonous influence on any of the higher animals even in extremely concentrated solutions. Following out numerous laboratory experiments by Dr. Klein on the destruction of microbes by a solution of 1 in 200 of this fluid (or rather emulsion), Bruce Clarke has made use of it as a surgical disinfectant in the following cases: (1) Fresh operation wounds uncomplicated by ulcers, sinuses, &c.; (2) ulcers, &c., demanding special purification, in which Thiersch's method of skin grafting was employed; (3) abscesses and sinuses, either septic or containing pus; (4) sinuses in connection with mucous membranes; (5) foul mucous membranes, cystitis, &c. From his practical experience, and from some excellent results obtained, the antiseptic seemed to him likely to prove more efficacious practically than any at present known. It is very powerful, and neither irritates the hands of the surgeon nor the skin of the patient. Harrell[4] has employed acetanilid (antifebrin) as a dressing, in the form of a fine dry powder, for wounds in which there is extensive loss of tissue. Pain disappeared soon after its free application, and there was no pus in any of his cases. It does not irritate the skin, has no offensive odour, and will not poison by absorption.

The bacillus pyocyaneus produces not only blue, but also green and brown pus and any possible shade between these colours. Green suppuration has not become less frequent in spite of antisepsis and asepsis. Schimmelbusch[5] found this bacillus in seven out of ten cases of removal of the breast in Bergmann's clinic. According to Muehsam's investigations this bacillus is a frequent parasite upon the body, inhabiting the armpits, the anal fissure, the inguinal folds, &c. The greenish discolouration of hydropathic compresses and linen of those that perspire profusely are evidences of its presence. It does not exist in the air. Although it is not an invasive micro-organism, yet, besides its disgusting odour, it greatly increases the discharge, prevents healing by disturbing the growth of granulations and by getting down between them impedes the formation of new integument. Insidious and slow intoxication may also result from green suppuration.

The important work of Hægler on the surgical significance of dust is reviewed by Warbosse.[6] It shows that the important factor in atmospheric infection is dust. Although this contains the germs the danger of infection from air containing pathogenic microbes is much less than from other sources. Germs are less in number in the atmosphere of a room unagitated though unclean that in that of a comparatively clean room in a state of agitation. Agitation of the air and of dry discarded dressings should be avoided during operations or dressing of wounds, and all dust should be precipitated by charging the air with moisture. Schimmelbusch,[7] from experiments upon animals, has learned that a great number of so-called antiseptics, sublimate, carbolic, lysol, zinc chloride, potash, nitric and acetic acids, &c., have not the power of destroying the sources of infection in infected wounds, however powerful their parasiticidal property may be on dead infected objects. He placed the septic material in the wounds of the animals and immediately afterwards rinsed the wound with the antiseptic material. The fatal results in his experiments resulted from the rapid absorption of the virus from the wounds; the micro-organisms probably entering the tissue spaces very quickly and soon being out of the reach of disinfectants.

### Fractures and Dislocations.

Bardenheuer, L. R. Lane, Allis, T. W. Nunn, Frere, and others have recently insisted that to put up all injuries of the elbow at right angles is a dangerous routine practice, as shown by the number of cases of subsequent deformity. The question of treatment of fractures of the lower end of the humerus in the right-angled and in the extended position is alluded to by Wight[8]. In five cases recorded by him which had been treated in the extended position midway between complete extension and right-angled flexion, or by a nearly straight splint the resulting ankylosis of the elbow joint and the disability of the limb were very great. Operative measures were resorted to with good result. In four other cases Wight used "forcible joint infraction" to remedy the ankylosis, two of these had been treated by the above method, one by a right-angled splint, and one only by a fully extended straight splint. In one other case treated by a nearly straight splint the limb was very much disabled. Further treatment by operation was declined in this case. Wight recommends the treatment of all cases with the forearm in the right-angled position on account of the stiffness of the elbow which so frequently results. Although, he says, there is the possibility of the displaced fragments being completely reduced when the arm is extended, yet, in order to obtain a useful limb, it may be permissible for the surgeon to leave the limb with some deformity. On the other hand, J. E. Moore[9] advises the straight position in the treatment of fractures involving the elbow joint. He believes that in the majority of cases the fragments can be controlled best in this position, and that the "carrying function" can be preserved. This function is produced by the forearm making an angle outwards with the arm when in extension. When the forearm is flexed it is very difficult to tell whether this function is preserved or not. If the fragments are so misplaced that they cannot be reduced by manipulation, they should be cut down upon under proper aseptic precautions and wired. The arm should be kept at rest for four or five weeks in a plaster of Paris splint until union is complete, when passive motion should be employed. The dressing should, however, be changed from time to time. Moore believes that many ankylosed joints following fracture are due to ill-advised passive motion rather than to the injury. If ankylosis should occur with the arm straight, it is a comparatively simple matter to be remedied. Meyer (W.) brought before the New York Surgical Society[10] a case of old ununited intra-capsular fracture of the neck of the femur treated by nail fixation. The patient was a man thirty-nine years of age, who had been crippled ever since a fall from a height of sixteen feet ten months previously. All the usual symptoms were present. After opening the hip joint by Langenbeck's incision, the ends of the two fragments were scraped free of all uniting connective tissue. The atrophied neck was then shaped to correspond to the concave fractured surface of the head, and fastened to it by two long nails, such as Wyeth recommends for resection of the knee joint. The shortening was found at the end of ten weeks to have been reduced from 3¼ to 1½ inches, and perfect bony union had apparently taken place between the fragments. Typical extra-capsular fracture of the neck of the femur, according to E. H. Bennett,[11] can result from muscular action without a fall or blow on the great trochanter. In support of this he quotes the first illustration in Sir Astley Cooper's "Dislocations," and a clinical example of his own. He also figures several exceptional specimens, in all of which the great trochanter has not been broken, and the lower fragment is to some degree impacted into the upper. The necessity of relaxing all the muscles of the leg in the treatment of Dupuytren's fracture is insisted upon by M. Duplay.[12] To do this he extends the foot, flexes the leg upon the thigh, and then flexes the thigh upon the pelvis. Reduction can now be effected (with or without chloroform) by grasping the dorsum of the foot with one hand and the heel with the other, while an assistant makes counter-extension by grasping the leg. The limb is then put up in plaster for six or eight weeks. Section of the tendo Achilles, he believes, is useless. In recent irreducible Dupuytren's fracture, the tibio-tarsal joint should be opened and the interposed fragments or other cause of irreducibility removed. It may be necessary in some cases to excise the joint, or even remove the astragalus. In old fractures united in bad position, M. Duplay recommends osteoclasia under chloroform, osteotomy of fibula, osteotomy of fibula with removal of wedge from tibia, or, lastly, tibio-tarsal resection without sacrificing the malleoli.

In treating fractures of the leg, Bergholf says[13] the latter should never rest on the bedding, but should be suspended. If the limb rest upon the bed each movement of the body is bound to move the fracture.

The treatment of fractures involving the joints is ably discussed by Klemm[14]. He notes the pathological changes which take place in the joint and surrounding important structures as the result of intra and par-articular extravasations of blood, and the prognosis dependent upon these conditions. Puncture of the hæmatoma, with or without subsequent irrigation, is recommended where the extravasation of blood is so large that gangrene is to be feared. Incision or suturing of bony fragments is to be reserved for cases

Intra-osseous splint in situ.

where the parts are already injured, or where reposition and coaptation of the fragments is impossible by other methods. Massage and compression, combined with active and passive motion, fill a most important *rôle* in the treatment of joint fractures, but reliance upon massage and motion with an entire absence of splints and immobilisation is certainly wrong. A simple method of treating compound fractures is adopted by Treves[15]. After the limb has been carefully cleansed with carbolic lotion, loose or damaged bone removed, displaced fragments adjusted, ordinary well-padded splints are applied by means of fine webbing and buckles, instead of strapping and bandages. The limb is kept exposed to the open air, being covered by a heap of iodoform or creolin, which keeps out the bacteria without hindering the free escape of discharges. These form with the antiseptic power a harmless scab or crust, and the damaged part is open to view.

Senn[16] introduces a new method of direct fixation of

Transverse fracture of humerus unmobilized by a wide perforated bone ferrule.

the fragments in the treatment of compound fractures and in that of simple fractures, in which for any reason, such as non-union, it is desirable to expose the fracture by incision. Intra-osseous tubes of sterilised absorbable bone are used; one end of the cylinder which is perforated is inserted into the medullary canal of one fragment, and the other end of it into the other. The early formation of intermediate callus is not interfered with, and the lumen of the cylinder is soon filled with this bone-producing material, the perforations in the tube allowing new blood-vessels to penetrate from the medullary cavity to the osseous shaft. The cylinders are made from the long bones of turkeys, rabbits, and chickens. Another method is by the use of a bone ferrule slipped over the fragments in the case of very oblique fractures, care being taken not to disturb the periosteum more than is necessary. These ferrules are made from the femur or tibia of the ox, according to the bone to be operated on. If larger than an inch they should be perforated. Sterilisation is effected by boiling for an hour or more, after which the rings are kept immersed in sublimate alcohol (1 to 1,000) ready for use. A plaster of Paris splint is used to fix the limb which can be fenestrated, if necessary, for drainage, &c. Senn has adopted this method in three cases with success.

In the case of ununited fractures of the shafts of long bones, especially in the lower extremity, W. Arbuthnot Lane[17] removes a good thick slice off the end of each fragment, so as to expose the normal structure of the shaft. The sawn surfaces he retains immovably in accurate position by employing screws instead of wire. The use of two screws completely obviates any rotation of the bony surfaces. Gowan's osteotome is an invaluable instrument, not only for ensuring the accuracy of parallelism in the sectional planes, but also for firmly holding the fragments during the boring and driving in of the screws. The screws produce no irritation, and do not require to be removed. In Mr. Lane's experience such conditions of non-union of fractures in the leg are always the result of the application of the principle of the vertical foot piece, the fallacy of which he has dealt with in a recent article.[18] In solutions of continuity of the leg and thigh, he shows experimentally the results of placing the lower segment in a position of inward rotation through an angle of 45 degrees, while the upper fragment and thigh are in the position of rest—viz., rolled outwards in the supine position. This rotation produces a considerable gaping of the fractured surfaces, and a consequent shortening of the limb due to the winding up and alteration in the direction of the muscles surrounding the broken bone. This shortening of the muscles would in a recent fracture be much increased by hæmorrhage into and beneath the muscles. In practice some of the deformity which ought to result from the vertical foot-piece is sometimes obviated by the foot slipping away from the vertical position, and by the outward rotation of the upper segment of the limb being limited to some extent by the limb being slung. In any solution of continuity of segments of the lower extremity the surfaces of the bone must be kept parallel and in apposition by ensuring that the axis of the lower segment shall correspond accurately with that of the upper; the shortening due to hæmorrhage and to the contraction of the muscles must be met by extension.

[1] Brit. Med. Journ., Jan. 28, Feb. 11, Feb. 18, 1893. [2] New York. Med. Journ., Dec. 2, 1893, p. 666. [3] Lancet, July 1. 1893. [4] Medical News, Phila,, Oct. 14, 1893, p. 438. [5] Sammlung Klinischer Vorträge, N.F., No. 62, 1892; Annals of Surgery, Oct. 1893. [6] Boston Med. and Surg. Journ., Med. Rec., Dec. 2, 1893. p. 734. [7] Verhandlungen der Deutsch, Gesellschaft fur Chirurgie, XXII., Korgess, 1893; Annals of Surgery, Oct., 1893. [8] Annals of Surgery, Aug., 1893. [9] Med. Record, N.Y., Nov.4, 1893, p.583. [10] Annals of Surgery, July, 1893, p. 30. [11] Dublin Journ. of Med. Science, Oct. 1893, p. 281. [12] Gazette des Hopitaux, July 6, 13, 1893; Q.M.J., Oct. 1893. [13] Med. Record, Nov. 11, 1893, p. 633, [14] Sammlung Klinisch Vorträge, No. 78, Sept. 1893; Annals of Surgery, Dec., 1893, p. 668. [15] Annals of Surgery, Nol. XVII., No. 2, February, 1893. [16] Annals of Surgery, Aug. 1893, p. 125. [17] Lancet, Dec. 16, 1893, p. 1501. [18] Brit. Med. Journ., Nov. 11, 1893, p. 1049.

## Figures and Tables

**Figure f1:**
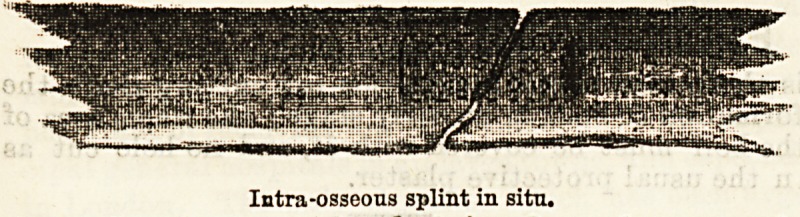


**Figure f2:**